# ESI(+)-MS and GC-MS Study of the Hydrolysis of *N*-Azobenzyl Derivatives of Chitosan

**DOI:** 10.3390/molecules191117604

**Published:** 2014-10-30

**Authors:** Fernanda S. Pereira, Heliara D. L. Nascimento, Alviclér Magalhães, Martin G. Peter, Giovana Anceski Bataglion, Marcos N. Eberlin, Eduardo R. P. González

**Affiliations:** 1 Laboratório de Química Orgânica Fina—C.P. 467, Programa de Pós-Graduação em Ciência e Tecnologia de Materiais (POSMAT), Departamento de Física, Química e Biologia, Campus de Presidente Prudente, Universidade Estadual Paulista, Presidente Prudente 19060-900, Brazil; E-Mail: fernanda.lqof@gmail.com; 2 Laboratório ThoMSon de Espectrometria de Massas, Instituto de Química, Universidade de Campinas UNICAMP, Campinas 13083-970, Brazil; E-Mails: heliara1@hotmail.com (H.D.L.N.); bataglion@hotmail.com (G.A.B.); eberlin@iqm.unicamp.br (M.N.E.); 3 Departamento de Química Inorgânica, Instituto de Química, Universidade de Campinas UNICAMP, Campinas 13083-970, Brazil; E-Mail: alvicler@iqm.unicamp.br; 4 Institut für Chemie, Universität Potsdam, Potsdam D-14476, Germany; E-Mail: martin.peter@uni-potsdam.de

**Keywords:** chitosan, *N*-azobenzylchitosan, ESI-MS, GC-MS, S_n_Ar reaction

## Abstract

New *N*-*p-*chloro-, *N*-*p-*bromo-, and *N*-*p-*nitrophenylazobenzylchitosan derivatives, as well as the corresponding azophenyl and azophenyl-*p*-sulfonic acids, were synthesized by coupling *N*-benzylvchitosan with aryl diazonium salts. The synthesized molecules were analyzed by UV-Vis, FT-IR, ^1^H-NMR and ^15^N-NMR spectroscopy. The capacity of copper chelation by these materials was studied by AAS. Chitosan and the derivatives were subjected to hydrolysis and the products were analyzed by ESI(+)-MS and GC-MS, confirming the formation of *N*-benzyl chitosan. Furthermore, the MS results indicate that a nucleophilic aromatic substitution (S_n_Ar) reaction occurs under hydrolysis conditions, yielding chloroaniline from *N*-*p-*bromo-, and *N*-*p-*nitrophenylazo-benzylchitosan as well as bromoaniline from *N*-*p-*chloro-, and *N*-*p-*nitrophenylazobenzyl-chitosan.

## 1. Introduction

Most azobenzenes can be isomerized from the *trans* to the *cis* configuration by UV-irradiation. After subsequent thermal relaxation, the molecule returns to its *trans* state. This characteristic is important in the study of azo-polymers as promising materials for technological applications such as optical information storage devices [1,2,3].

Several azo compounds have also shown biological and pharmaceutical activities such as antibacterial [4], antiviral [5], antifungal [6], antitumor [7,8,9], and anti-inflammatory properties [10]. Moreover, azo-polymers are employed with the aim of selective drug release. The use of aromatic azo-polymers as the trigger for colon-specific drug delivery based on enzymatic reduction and subsequent splitting of the azo bond in the large intestine to release the drug has therefore been reported [11].

Scientists have shown great interest in chitosan in pharmaceutical and biomedical applications due to its biological properties such as biocompatibility, biodegradability, nontoxicity and low-immunogenicity. The presence of reactive groups in the polymer offers great opportunity for chemical modification, which leads to a wide range of derivatives, such as azo-polymeric compounds, potentially suited for drug delivery applications. The linkage between the polymer and the drug can be cleaved by hydrolysis or enzymatic degradation at the target site where the drug could be released.

Furthermore, chitosan and some of its derivatives have been used in wastewater treatment due to their ability to coordinate with transition metal ions. Azobenzene chitosan compounds were previously reported as metal chelating materials for gold, platinum and palladium [12,13]. Moreover, the copper complexes of chitosan and its derivatives were reported as materials in different fields such as catalysis [14,15,16] and in medicines with antibacterial [17,18] and antitumor properties [19].

In the present work, we report the synthesis and characterization of the new chitosan derivatives: poly-*N*-(4-(4-*R*-chlorophenyldiazenyl)benzyl)-chitosan (**1**), poly-*N*-(4-(4-*R*-bromophenyldiazenyl)-benzyl)-chitosan (**2**), poly-*N*-(4-(4-*R*-nitrophenyldiazenyl)benzyl)-chitosan (**3**), poly-*N*-(4-(4-*R*-benzyl-chitosanphenyl)diazenyl)benzenesulfonic acid (**4**) and poly-*N*-(4-(4-*R-*phenyldiazenyl)benzyl)-chitosan (**5**), which could be of interest in further studies as simulation systems for biological applications.

Preliminary studies of the chelating capacity of these materials with Cu(II) were performed by atomic absorption spectrophotometry. The depolymerization of chitosan and derivatives facilitates the characterization and the study of the polymer properties. Therefore, to confirm formation, these products were hydrolyzed by means of 10 M aqueous hydrochloric acid and the resulting products were analyzed by ESI(+)-MS and GC-MS. This work also aims to investigate if the experimental procedure used is suitable to prepare such azo-compounds. *N*-benzyl chitosans can be used as efficient diazonium salts trapping due the moderate conditions required for azo-coupling reaction and easy work up.

## 2. Results and Discussion

**Scheme 1:**
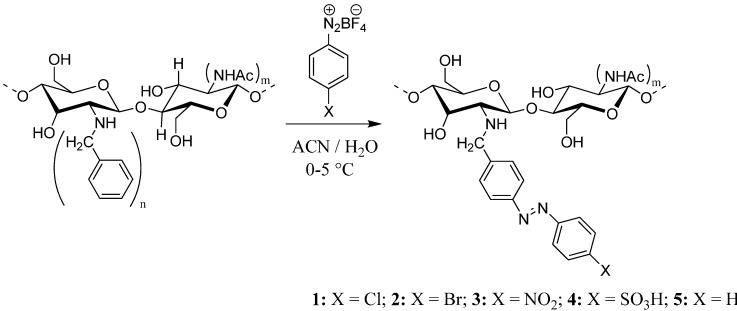
Synthesis of *N*-phenylazobenzyl chitosans.

The *N*-azobenzylchitosan compounds were synthesized via azo-coupling of *N*-benzylchitosan and the corresponding *p*-substituted benzenediazonium tetrafluoroborate salts (Scheme 1).

**Figure 1:**
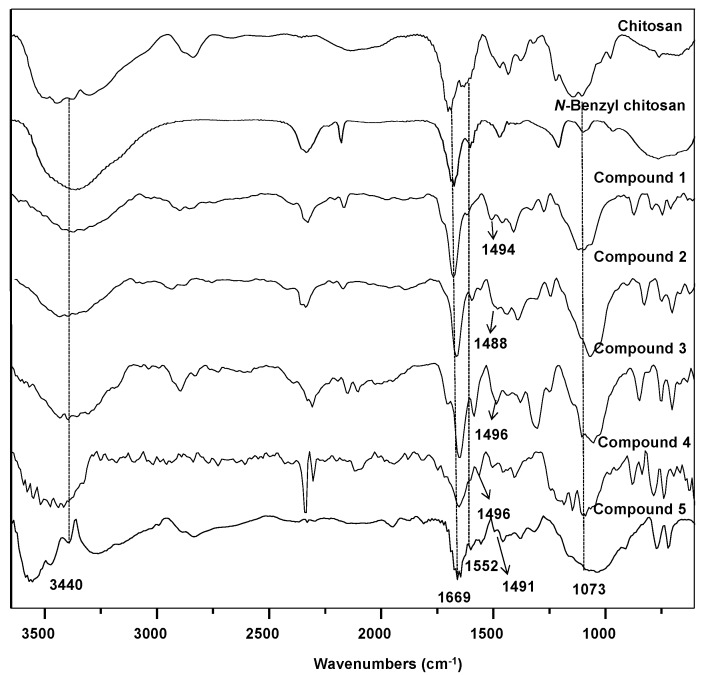
FT-IR spectra of chitosan, *N*-benzyl chitosan and compounds **1**–**5**.

In comparison with chitosan, the FT-IR spectrum of *N*-benzyl chitosan shows additional bands at 1552 cm^−1^ ν(N-H) and ν(C=C), which are absorptions characteristic of this compound [20]. For the *N*-azobenzyl chitosan derivatives, a band in the 1436–1496 cm^−1^ region for ν(N=N) and the bands of the individual functional groups are observed, which confirm their formation. The FT-IR spectra are shown in Figure 1.

The ^1^H-NMR analysis of the *N*-azobenzyl derivatives shows additional signals in the range between 7.30 and 8.30, which suggest the presence of another aromatic ring [21]. The signals between 1.85 and 5.06 ppm correspond to the GlcNAc sugar units as well as azobenzyl substituted and unsubstituted GlcN. The ^1^H-NMR spectra of all the compounds can be found in the Supporting Information.

**Figure 2:**
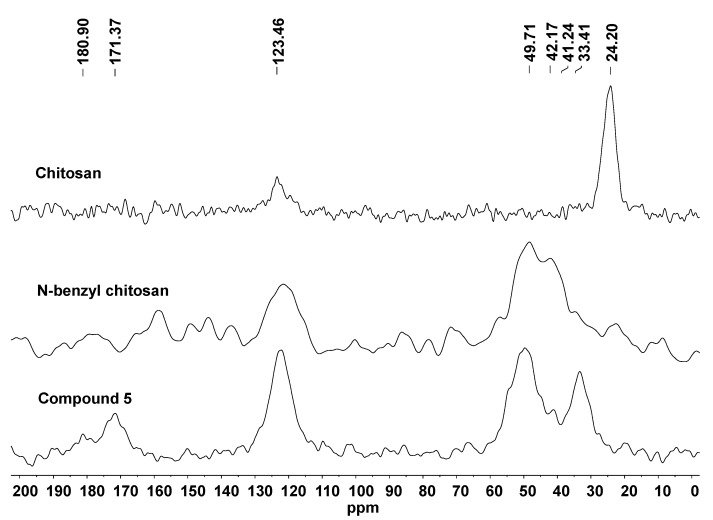
^15^N-NMR spectra of chitosan, *N*-benzyl chitosan and compound **5**.

The ^15^N-NMR spectrum of chitosan shows two signals at 24.20 and 123.46 ppm related to the amino group at C-2 and to the *N*-acetylated group, respectively [22]. For *N*-benzyl chitosan, the spectrum displays an additional signal at 42.17 ppm which corresponds to its benzylated-NH groups. The ^15^N-NMR spectrum of **5** shows a reduction in the signal area intensity at 41.24 ppm, attributed to the benzylated-NH which did not yield a coupling product with diazonium salts. On the other hand, an additional signal is observed at 33.41 ppm, which corresponds to benzylated-NH groups that were transformed to an azo-compound by a coupling reaction with *p*-substituted-benzenediazonium salts. Furthermore, two other signals appear in the spectrum at 180.90 and 171.37 ppm, which are attributed to -N=N- groups present in the azo-compound. The ^15^N-NMR spectra of the compounds are displayed in Figure 2.

The degree of substitution (DS) was determined by ^15^N-NMR technique [23]. The values obtained were 56% for *N*-benzyl chitosan and 42% for **5**. For comparison, the DS values were also calculated by ^1^H-NMR [24]. *N*-benzyl chitosan was obtained in 50%, **1** in 66%, **2** in 31%, **3** in 51%, **4** in 52% and **5** in 46% yield. The DS values for *N*-benzyl chitosan and **5** were quite similar for both techniques.

Chitosan and the derivatives were depolymerized by means of 10 M HCl in order to characterize the products by ESI(+)-MS. The chitosan hydrolysates contain GlcN oligomers up to DP5 (Table 1).

**Table 1:** Calculated *m*/*z* of GlcN oligomers and *m*/*z* values observed by ESI(+)-MS of chitosan hydrolysates.

Oligomers	Calcd. *m*/*z* [M + H]^+^	Found *m*/*z* [M + H]^+^	Found *m*/*z* [M-H_2_O + H]^+^
GlcN	180.06	180.16	162.14
GlcN_2_	341.06	341.29	323.29
GlcN_3_	502.06	502.82	484.82
GlcN_4_	663.06	663.57	645.28
GlcN_5_	824.06	824.70	-

**Figure 3:**
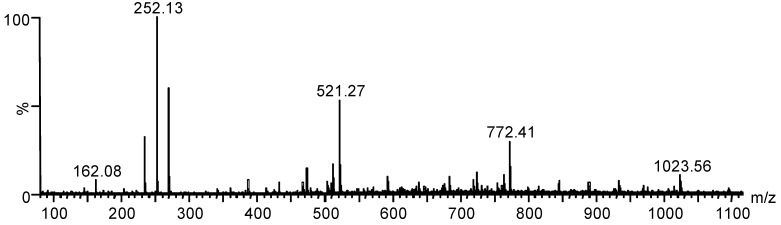
ESI(+)-MS from the hydrolysis reaction mixture of *N*-benzyl chitosan (10 M HCl).

ESI(+)-MS results of *N*-benzyl chitosan hydrolysates showed that the *N*-alkylation reaction is confirmed by increments of 90 *m*/*z* units in relation to the respective values of *m*/*z* of chitosan oligosaccharides. The oligomers of *N*-benzyl chitosan formed during acid hydrolysis and their *m*/*z* values are shown in Figure 3 and listed in Table 2.

**Table 2:** Values for *m/z* and their structural assignments for products formed during *N*-benzyl chitosan acid hydrolysis.

Ion	*m/z* Calcd.	*m/z* Found
[GlcN-H_2_O + H]^+^	162.06	162.08
[GlcNBn-H_2_O + H]^+^	252.06	252.13
[(GlcNBn)_2_ + H]^+^	521.06	521.27
[(GlcNBn)_3_ + H]^+^	772.06	772.39
[(GlcNBn)_4_ + H]^+^	1023.06	1023.54

**Figure 4:**
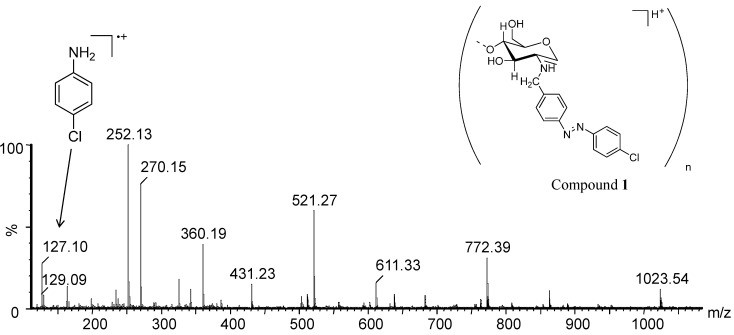
ESI(+)-MS from the hydrolysis reaction mixture of **1** (10 M HCl).

For the *N*-azobenzyl chitosan compounds, the acid hydrolysis reaction decomposed these derivatives due to the high acidity of the reaction medium and the temperature. As a consequence of this decomposition, the expected ions were not observed in the MS spectra. Figure 4 shows the ESI(+)-MS spectrum of the products formed during the acid hydrolysis reaction of **1**.

**Figure 5:**
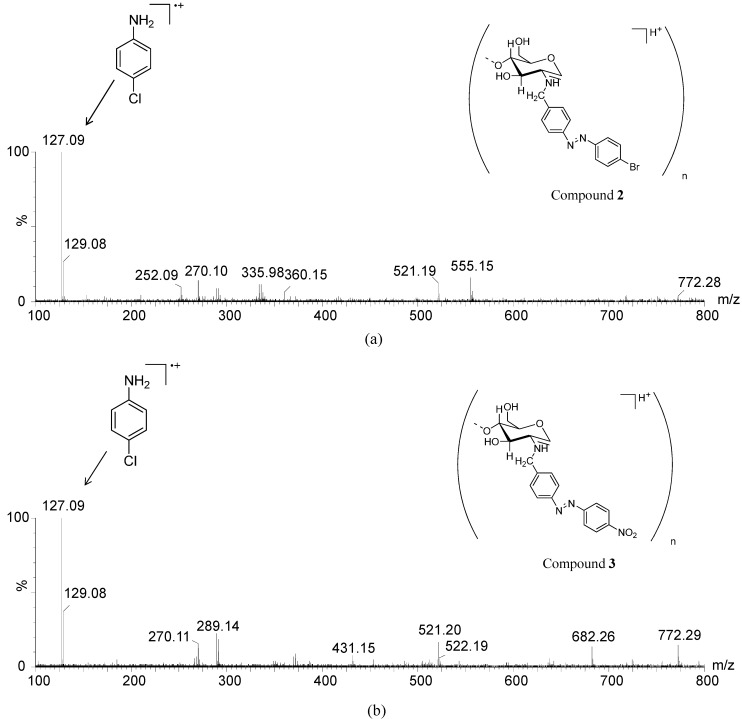
ESI(+)-MS from the hydrolysis reaction mixture of (**a**) compound **2** and (**b**) compound **3** (10 M HCl).

Besides the *m*/*z* values of *N*-benzyl chitosan oligomers, the MS spectrum also shows [M-H_2_O + H]^+^ ions which corresponds to the *p*-substituted aniline product from the azo compound decomposition in acid hydrolysis. This decomposition is likely promoted by the high concentration of H^+^ ions which facilitates the protonation of the azo group nitrogen atoms [25,26]. In **2** (Figure 5a), the ion product of *m*/*z* 127 with the isotopic pattern characteristic of the presence of chlorine atom (1:3 *m*/*z* 129/127) was observed, which corresponds to *p*-chloroaniline. It is therefore suggested that a nucleophilic aromatic substitution reaction (S_n_Ar) has occurred via which the bromine atom of **2** has been substituted by the chlorine atom. The substitution reaction likely happens because of the high concentration of chloride ions in the solution. However, the ion of *m*/*z* 171 attributed to *p*-bromoaniline is still observed due to incomplete substitution of bromine atoms. The S_n_Ar reaction was also observed for **3** (Figure 5b).

It can be assumed therefore that the ions of *m*/*z* 127 and 129 are not associated with *p*-chloroaniline impurities because such molecule was not used as a precursor in the synthesis of the diazonium salt, which was used instead of *p*-bromoaniline and *p*-nitroaniline to form **2** and **3**, respectively.

**Scheme 2:**
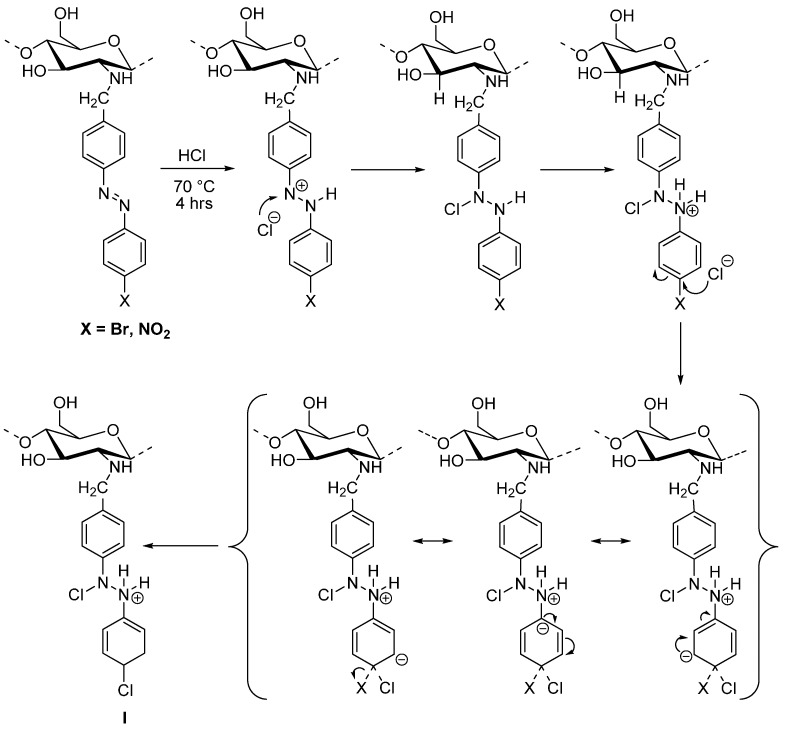
Proposal of a possible S_n_Ar mechanism for introduction of chlorine during acid hydrolysis of the modified polymer.

As a proposal for the detection of the ions of *m*/*z* 127 and 129 observed in Figure 5, Scheme 2 shows a possible S_n_Ar mechanism by protonation of the azo group of **2** and **3** and subsequent substitution of the bromine atom and nitro group [27,28] by a chlorine atom, leading during acid hydrolysis to a hypothetical intermediate **I**. A more extensive study of the hydrolysis process is being carried out and the results will be communicated in due course.

**Figure 6:**
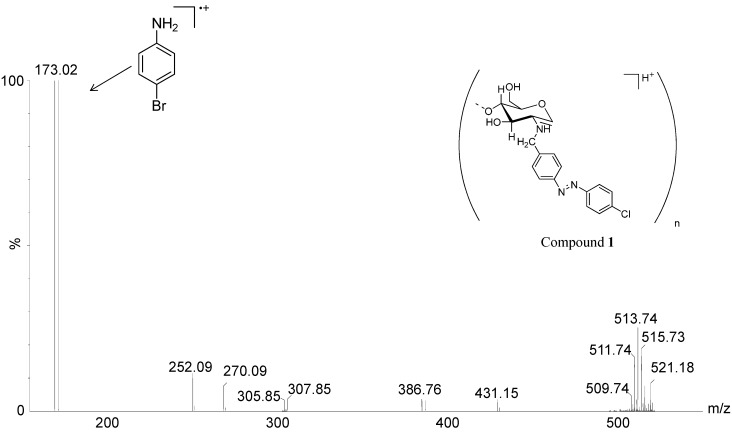
ESI(+)-MS for the hydrolysis reaction mixture of **1** (9 M HBr).

**Figure 7:**
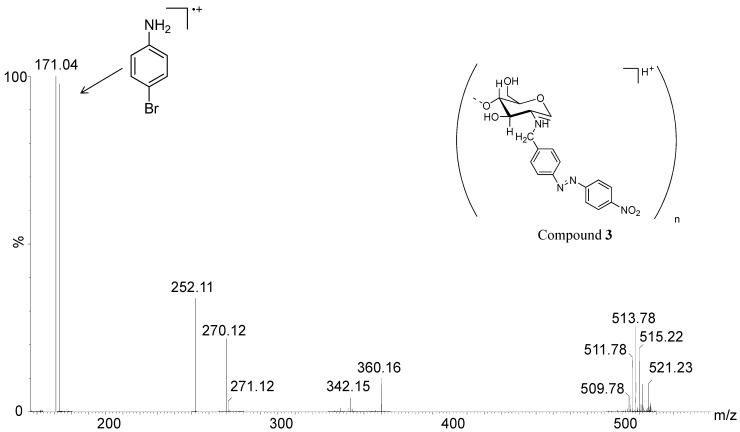
ESI(+)-MS for the hydrolysis reaction mixture of **3** (9 M HBr).

In order to confirm the S_n_Ar reaction, a hydrolysis was performed for **1** and **3** using 9 M HBr. ESI(+)-MS analysis of the respective hydrolysates showed in both cases the formation of *p*-bromoaniline products via the ions of *m*/*z* 171/173, which are the most abundant in the spectra (Figure 6 and Figure 7), apart from the ions of *m*/*z* corresponding to chitosan and *N*-benzyl chitosan oligomers. The apparent increase of S_n_Ar products, which were originated during hydrolysis with 9 M HBr, can be explained by the higher nucleophilicity of bromide ion. It can also be observed another hydrolysis product formed during the reaction, which corresponds to poly-bromoaniline confirmed by the ions of *m*/*z* 509/5011/513/515/521 with the isotopic pattern characteristic of the presence of bromine atoms, in both spectra [29,30,31].

To investigate whether the S_n_Ar reaction had taken place in residual diazonium salt or its aniline precursor, hydrolysis of *p*-bromobenzenediazonium salt and *p*-bromoaniline were carried out using 10 M HCl. GC-MS (70 eV and direct introduction) and analysis did not show any S_n_Ar products.

**Figure 8:**
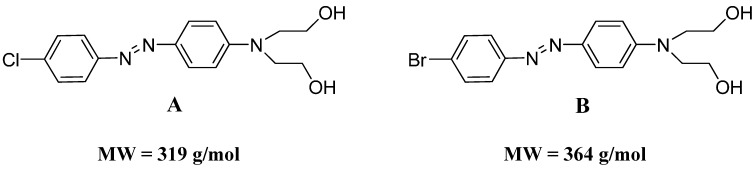
Structure of (**A**) 2,2'-(4-((4-chlorophenyl)diazenyl)phenylazanediyl)diethanol and (**B**) 2,2'-(4-((4-bromophenyl)diazenyl)phenylazanediyl)diethanol.

**Figure 9:**
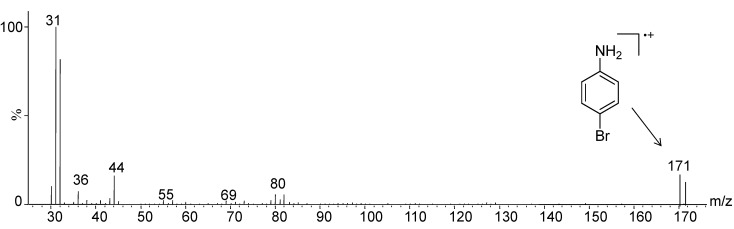
EI-MS of compound **A** detected via GC/MS analysis of the hydrolysis reaction mixture (9 M HBr).

**Figure 10:**
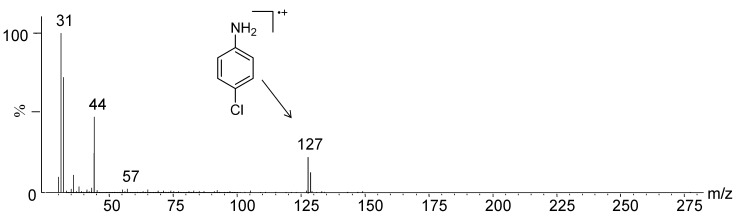
EI-MS of compound **B** detected via GC/MS analysis of the hydrolysis reaction mixture (10 M HCl).

In addition, an acid hydrolysis with 10 M HCl and 9 M HBr was carried out under the same experimental conditions used for *N*-azobenzyl chitosan derivatives hydrolysis, previously discussed, for two different azo dye compounds synthesized and characterized in our laboratory (Figure 8). Figure 9 and Figure 10 present the GC-MS results which confirm that the S_n_Ar reaction also happens for these compounds. These experiments corroborate the proposed S_n_Ar reactions during hydrolysis of *N*-azobenzylchitosan compounds and confirm their synthesis.

To investigate the chelating capacity of chitosan and derivatives, the synthesis of Cu(II) complexes was carried out at room temperature for 24 h and pH 6.5. The percentage of Cu^2+^ chelated and the corresponding milligram by gram of each compound are presented in Table 3. Chitosan has shown higher capacity with 75% of Cu^2+^ ions chelated.

**Table 3:** AAS results of copper content chelated by chitosan and derivatives.

Compounds	% of Cu^2+^ Chelated	mg/g of Cu^2+^ Chelated
Chitosan	75	120
*N*-benzyl chitosan	14	23
**1**	14	23
**2**	11	18
**3**	9	15
**4**	10	16
**5**	9	15

## 3. Experimental Section

### 3.1. General Information

Chitosan (M_r_ 75–160 KDa) was purchased from Sigma-Aldrich (São Paulo, Brazil). The degree of deacetylation was 78%, as determined by ^15^N-NMR [23] and ^1^H-NMR [24]. All reagents were of analytical grade and used without further purification. The FT-IR spectra were performed on a Model Vector 22 spectrophotometer (Bruker, Presidente Prudente, Brazil) using KBr pellets. Spectra were recorded at 23 °C in the 4000–400 cm^–1^ range at a resolution of 16 cm^−1^ and 120 scans. ^1^H-NMR analyses were recorded on an AVANCE III NMR spectrometer (Bruker, Campinas, Brazil, 400 MHz) at 25 °C. Solvents: D_2_O/0.1 M DCl for chitosan and *N*-benzylchitosan, and DMSO-*d*_6_ for the azobenzene derivatives. ^15^N-NMR experiments were recorded on AVANCE III NMR spectrometer (Bruker, Campinas, Brazil, 300 MHz) equipped with dual probe. CP/MAS experiments used a rotation of 10 KHz in MAS and the contact time was 5 ms. The time between experiments was 1 s. The UV-Vis spectra were recorded on a Model Cary 50 spectrophotometer (Varian, Presidente Prudente, Brazil) with quartz cuvettes. Range: 190–800 nm. Samples of 5 mg were dissolved in 10 mL of DMSO. ESI(+)-MS analyses were recorded on a Synapt HDMS time-of-flight high resolution mass spectrometer (Waters, Manchester, UK). Solutions in H_2_O–MeOH (1:1), containing *ca.* 0.1% of formic acid were infused directly into the ESI source by means of a syringe pump at a flow rate of 15 μL·min^−1^. The operation conditions were: capillary and cone voltages 2800 V and 30 V, respectively; desolvation temperature 200 °C. Analysis was carried out on GC-MS Shimadzu QP-2010 plus (Presidente Prudente, Brazil). Ionization energy: 70 eV. Interface temperature: 240 °C; ion source temperature: 300 °C; start time: 2.0 min; end time: 35.0 min; DI temperature program: initial temperature: 100 °C, heating rate: 20 °C/min until 350 °C, hold time: 10 min. AAS analyses was performed in an A Analyst 200—Atomic Absorption Spectrometer (PerkinElmer, Presidente Prudente, Brazil).

### 3.2. Reductive N-Alkylation of Chitosan

Chitosan (0.5 g) was dissolved in 1% acetic acid (15 mL). The solution was diluted with ethanol (30 mL) and stirred for 30 min to form a gel. The pH of the solution was adjusted to 4–5. After that, benzaldehyde (2 mmol) dissolved in ethanol (99%, 10 mL) was added to the solution and stirred for 8 h at 60 °C. Sodium cyanoborohydride (0.12 g, 2 mmol) dissolved in ethanol (99%, 10 mL) was added and the mixture was stirred at room temperature. After 24 h, the pH was adjusted to 7 with aqueous NaHCO_3_ (5%, 15 mL). The white precipitate was filtered off and washed with water and ethanol to remove the excess of reagents. Afterwards, the material was dried for 1 h at 60 °C, yielding 0.7 g of product.

### 3.3. General Procedure for Azo-Coupling

*p*-Substituted benzenediazonium tetrafluorborates (*p*-Cl 0.22 g, *p*-Br 0.26 g, *p*-NO_2_ 0.23 g, *p*-SO_3_H 0.27 g and *p*-H 0.19 g, 1 mmol) were dissolved in a minimal volume of acetonitrile or ~5 mL of water. *N*-benzylchitosan (0.5 g) was slowly added to the solutions and the mixture was stirred for 20 min at 0–5 °C. The colored products were filtered off, washed with ethanol and dried for 3 h at 35 °C. The products showed solubility in dimethyl sulfoxide (DMSO).

### 3.4. General Procedure for Acid Hydrolysis of Chitosan and Derivatives

Acid hydrolysis of chitosan and the derivatives was carried out according to the literature [32]. Hydrochloric acid (50 mL of 10 M HCl) or hydrobromic acid (50 mL of 9 M HCl) was added to of chitosan or a derivative (1 g) in a round bottom flask and the mixture was stirred for 4 h at 70 °C. Afterwards, the solvent was removed in a rotary evaporator to yield a brown solid.

### 3.5. General Procedure for Copper Complexes Formation

The complexes were prepared by adding chitosan or derivatives (0.1 g) to a CuSO_4_·5H_2_O aqueous solution (0.01 mol/L, 10 mL) with stirring at room temperature and pH 6.5 for 24 h. After that, the mixture was filtered and the precipitate was dried in an oven at 60 °C for 4 h.

## 4. Conclusions

The chemical modification of chitosan polymer for the synthesis of new *N*-azobenzyl chitosan derivatives was confirmed by ^1^H-NMR, ^15^N-NMR and FT-IR techniques. The UV-Vis analysis of such compounds exhibits intense absorption bands and the nitro derivative showed a red shifted maximum intensity. Mass spectrometry data of *N*-azobenzyl chitosan hydrolysates allowed us to propose a nucleophilic aromatic substitution reaction (S_n_Ar) via which the bromine atom and the nitro group were replaced by a chlorine atom, a reaction that was facilitated by high concentration of chloride ions in the reaction media. The S_n_Ar reaction was confirmed by the substitution of chlorine atoms and nitro group of **1** and **3**, respectively, by bromine atoms during acid hydrolysis of both of these compounds with 9 M HBr. In addition, the formation of **1** and **3** could be confirmed by the ESI-MS of the hydrolysates from 9 M HBr hydrolysis, which showed that the cleavage of the azo linkage did not occur in some oligomers. Moreover, no substitution was observed for the precursors *p*-substituted anilines or the corresponding aryl diazonium salts in the acid hydrolysis. The presence of the azo group therefore seems to be important to promote the S_n_Ar reaction. We note that such S_n_Ar reaction under the described hydrolysis conditions has not been previously reported. Although presenting lower chelating capacity, the materials can form complexes with copper ions and this ability can be further explored in catalytic systems and for biological applications.

## Supplementary Materials

Supplementary materials can be accessed at: http://www.mdpi.com/1420-3049/19/11/17604/s1.

## Supplementary Files

Supplementary File 1
