# Supplementary material for: ESI(+)-MS and GC-MS Study of the Hydrolysis of N-Azobenzyl Derivatives of Chitosan
Source: Molecules. 2014 Oct 30;19(11):17604–18. doi: 10.3390/molecules191117604 (PMC6271483; doi:10.3390/molecules191117604)

Supplementary Materials

Figure S1. FT-IR spectrum of *N*-benzyl chitosan.

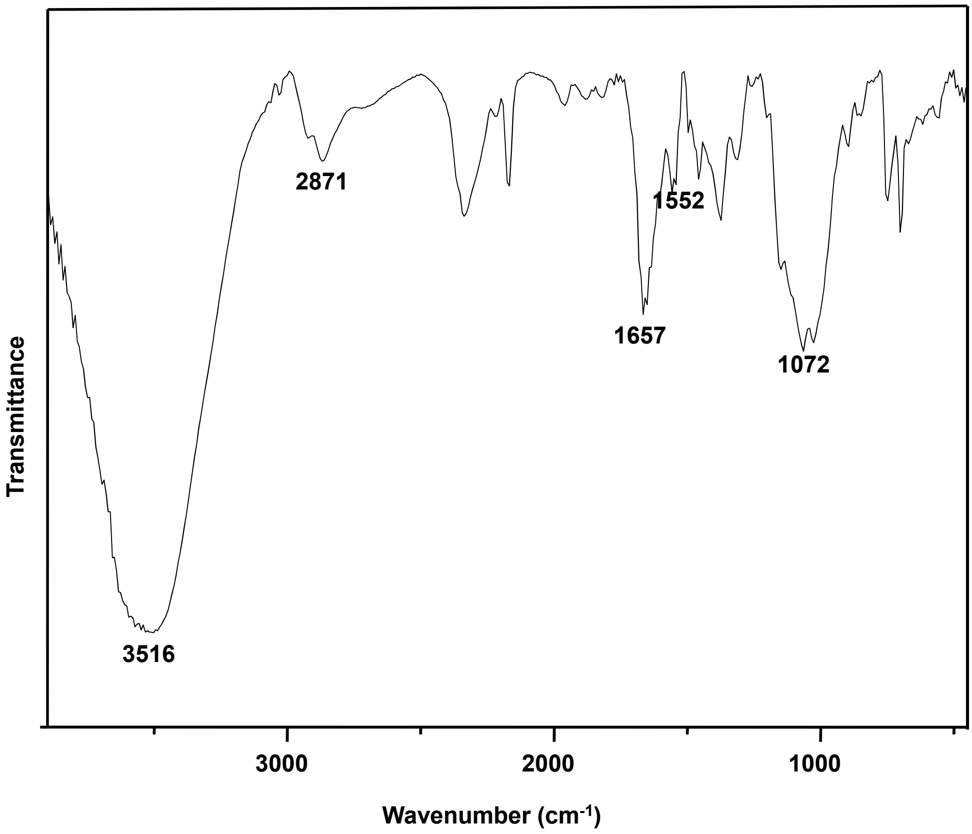

Figure S2. FT-IR spectrum of compound **1**.

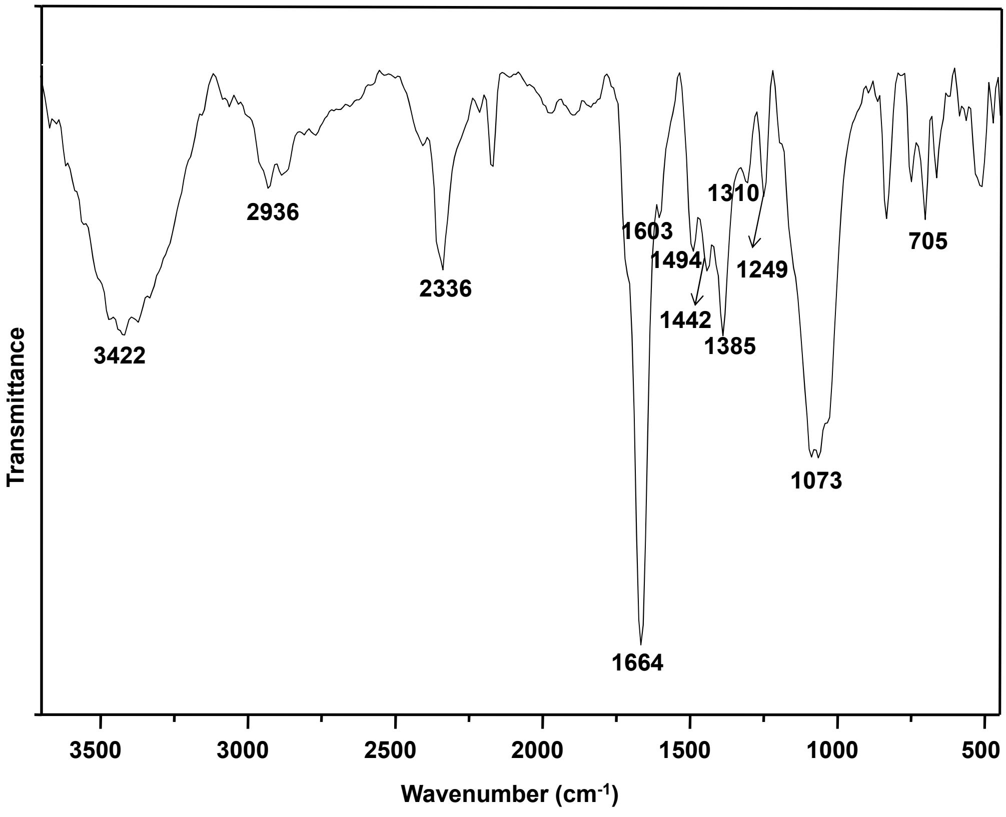

Figure S3. FT-IR spectrum of compound **2**.

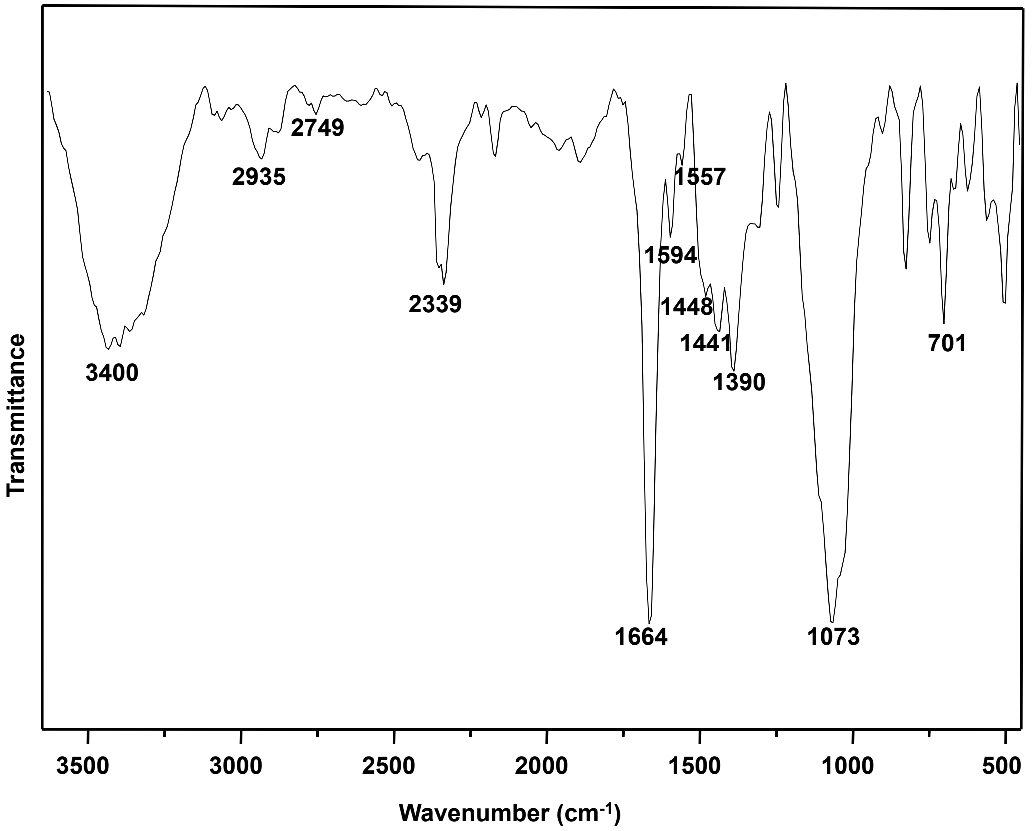

Figure S4. FT-IR spectrum of compound **3**.

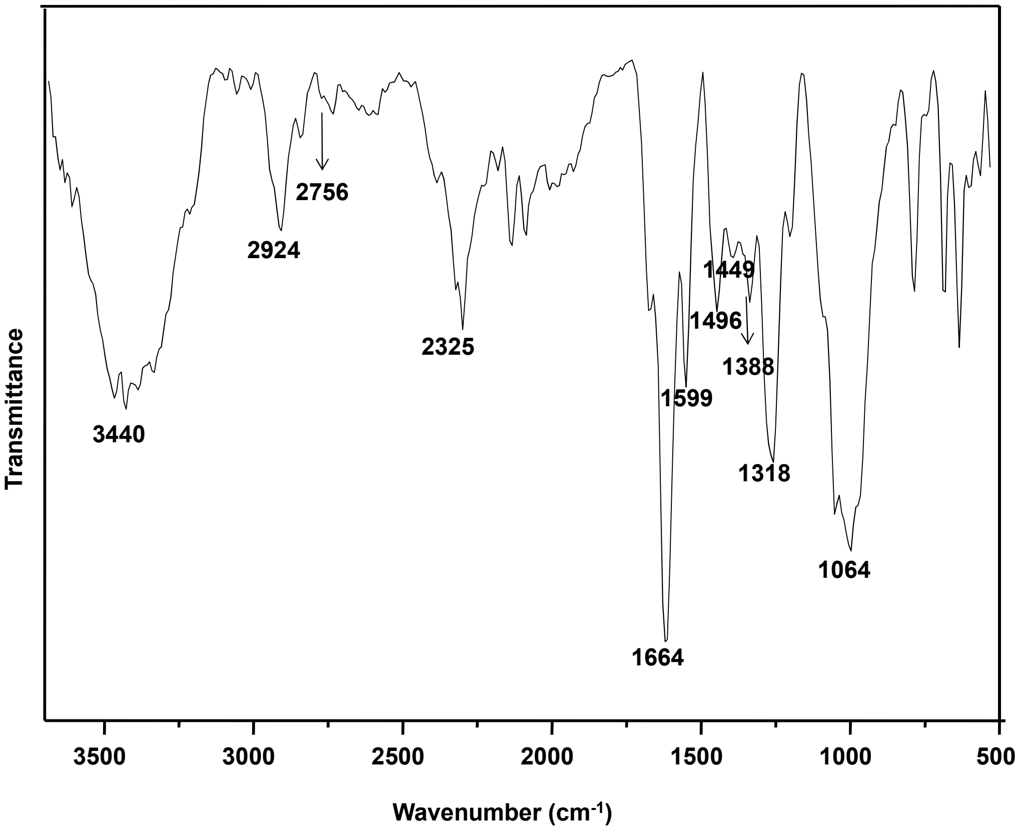

Figure S5. FT-IR spectrum of compound **4**.

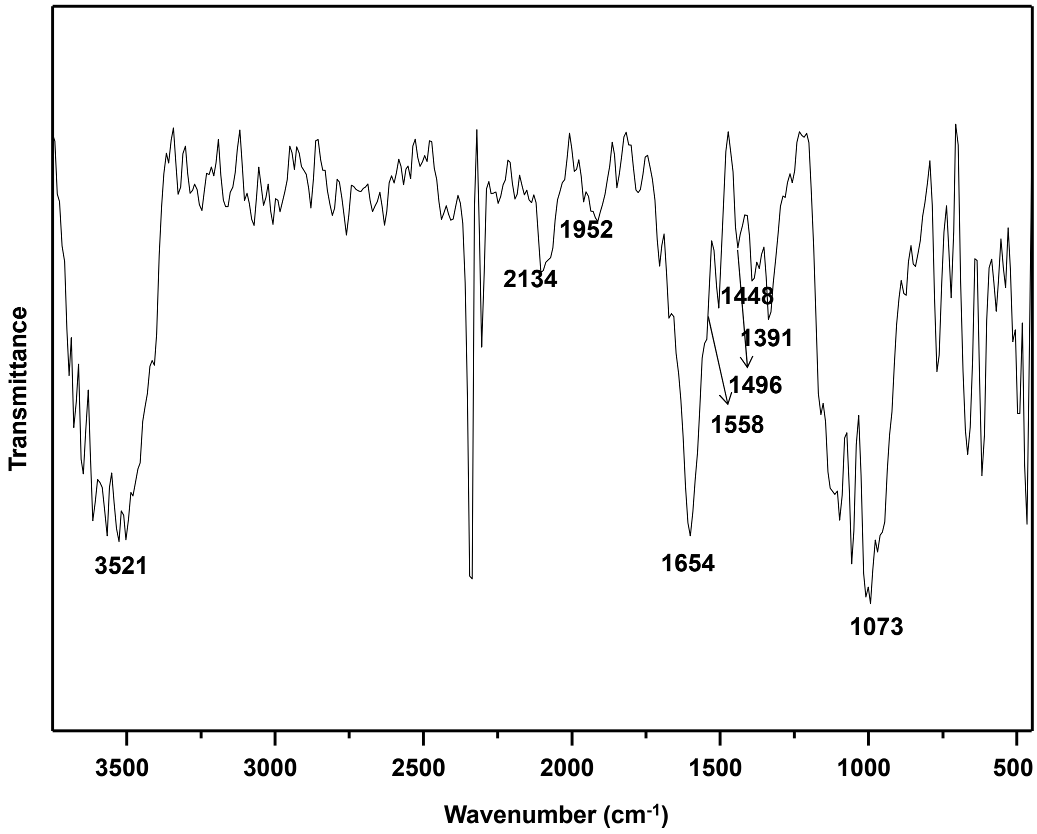

Figure S6. FT-IR spectrum of compound **5**.

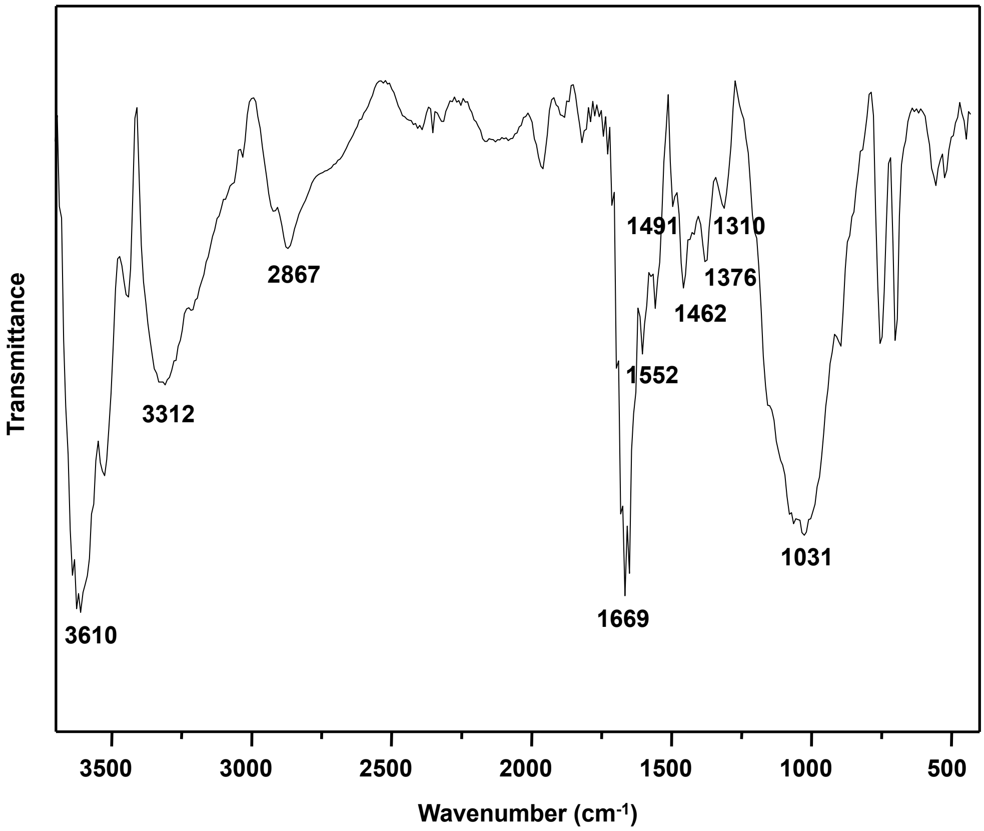

Figure S7. ^{1}H-NMR spectrum of chitosan.

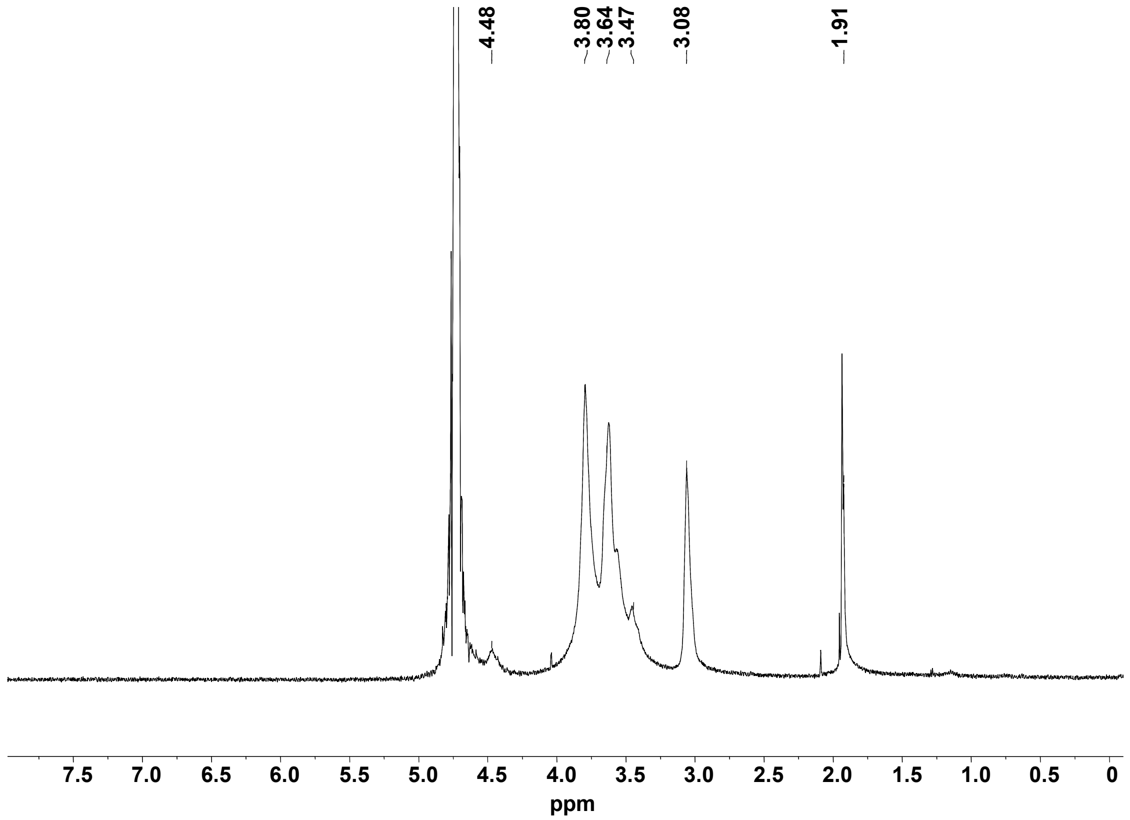

Figure S8. ^{1}H-NMR spectrum of *N*-benzyl chitosan.

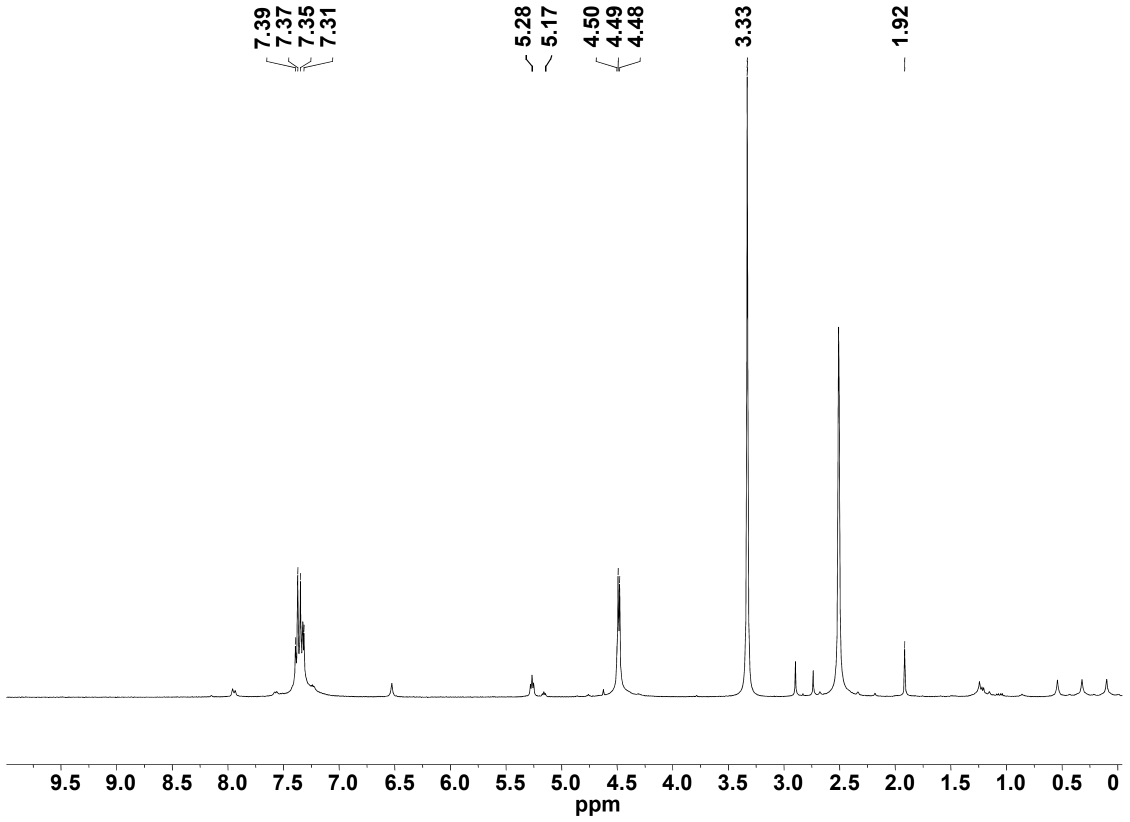

Figure S9. ^{1}H-NMR spectrum of compound **1**.

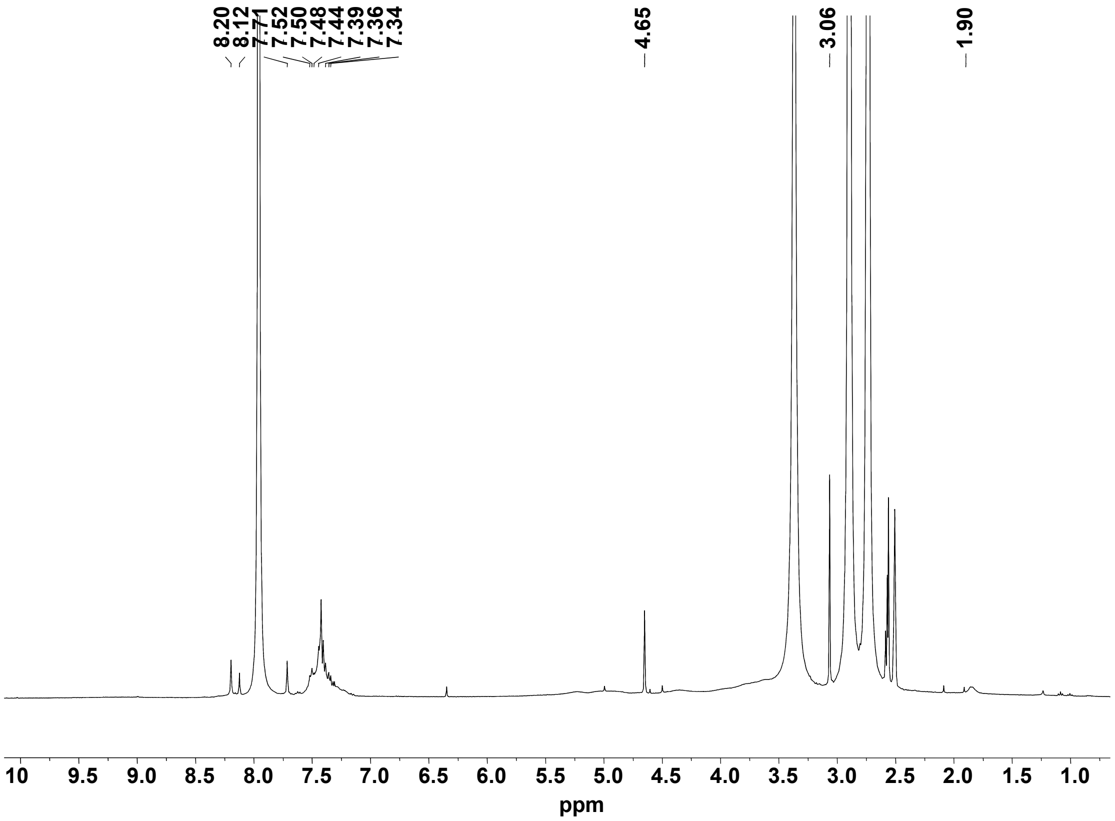

Figure S10. ^{1}H-NMR spectrum of compound **2**.

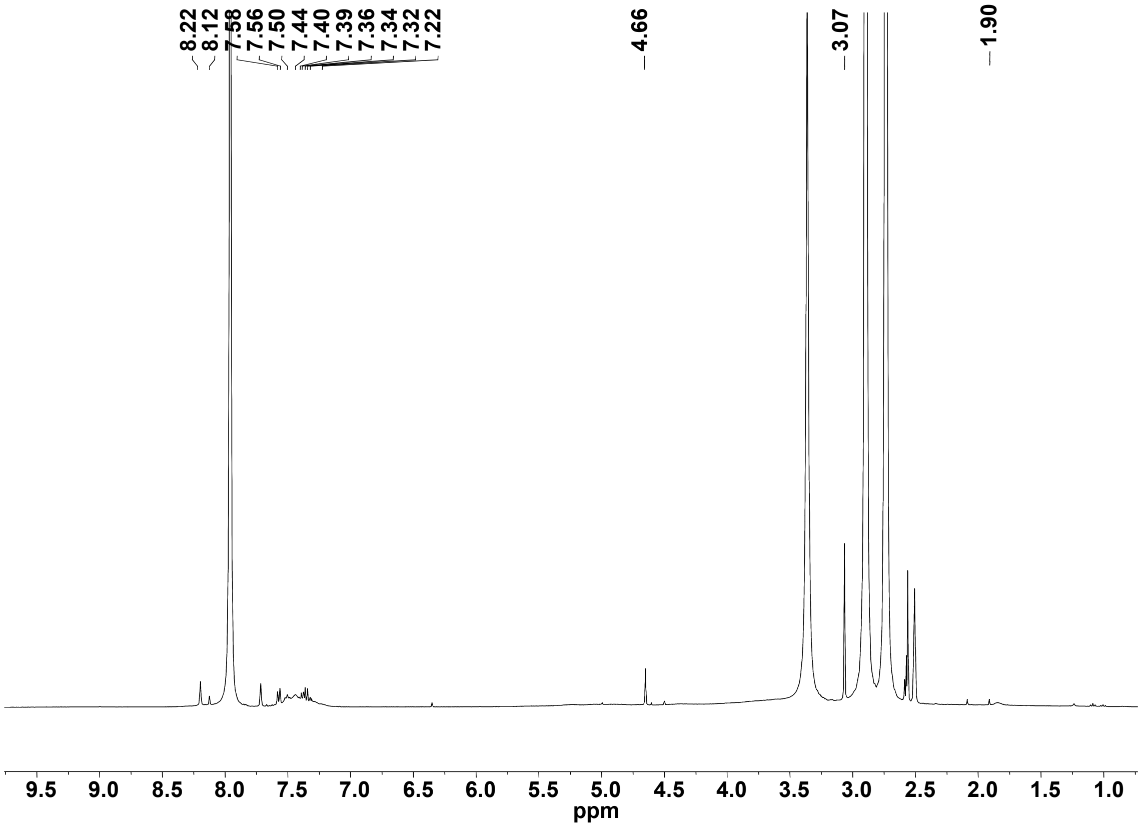

Figure S11. ^{1}H-NMR spectrum of compound **3**.

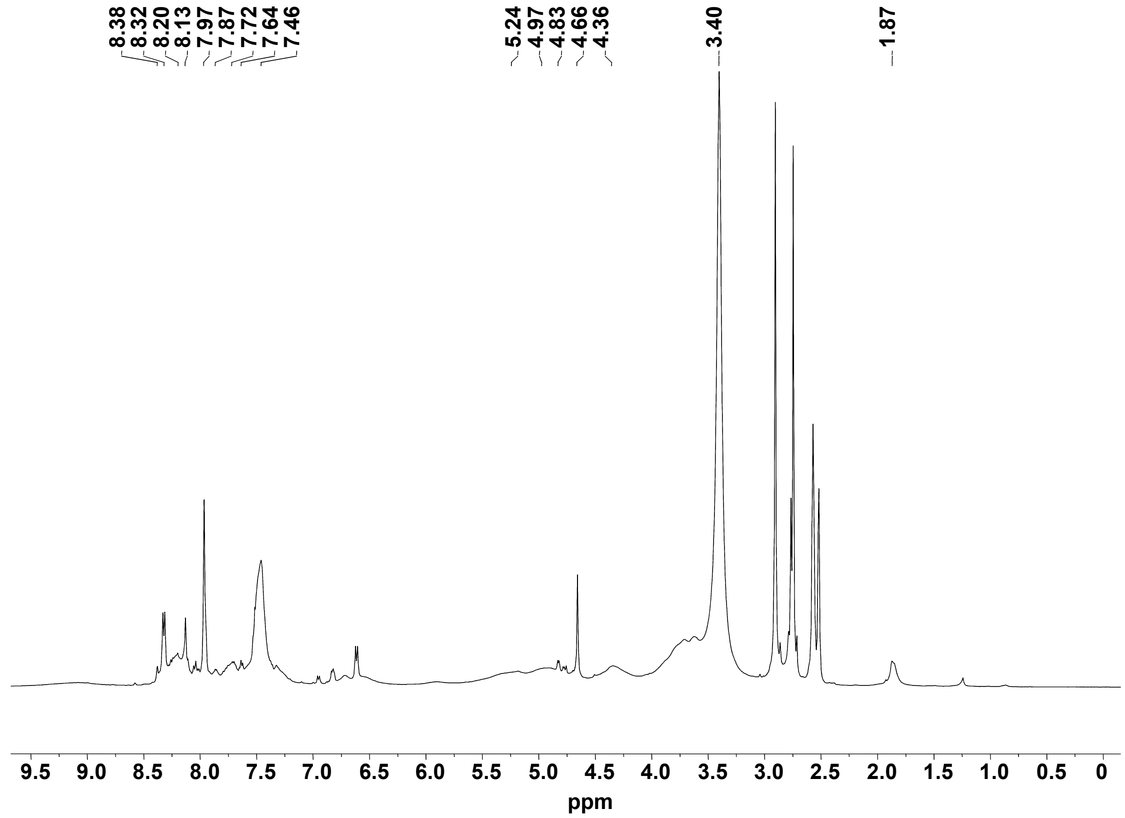

Figure S12. ^{1}H-NMR spectrum of compound **4**.

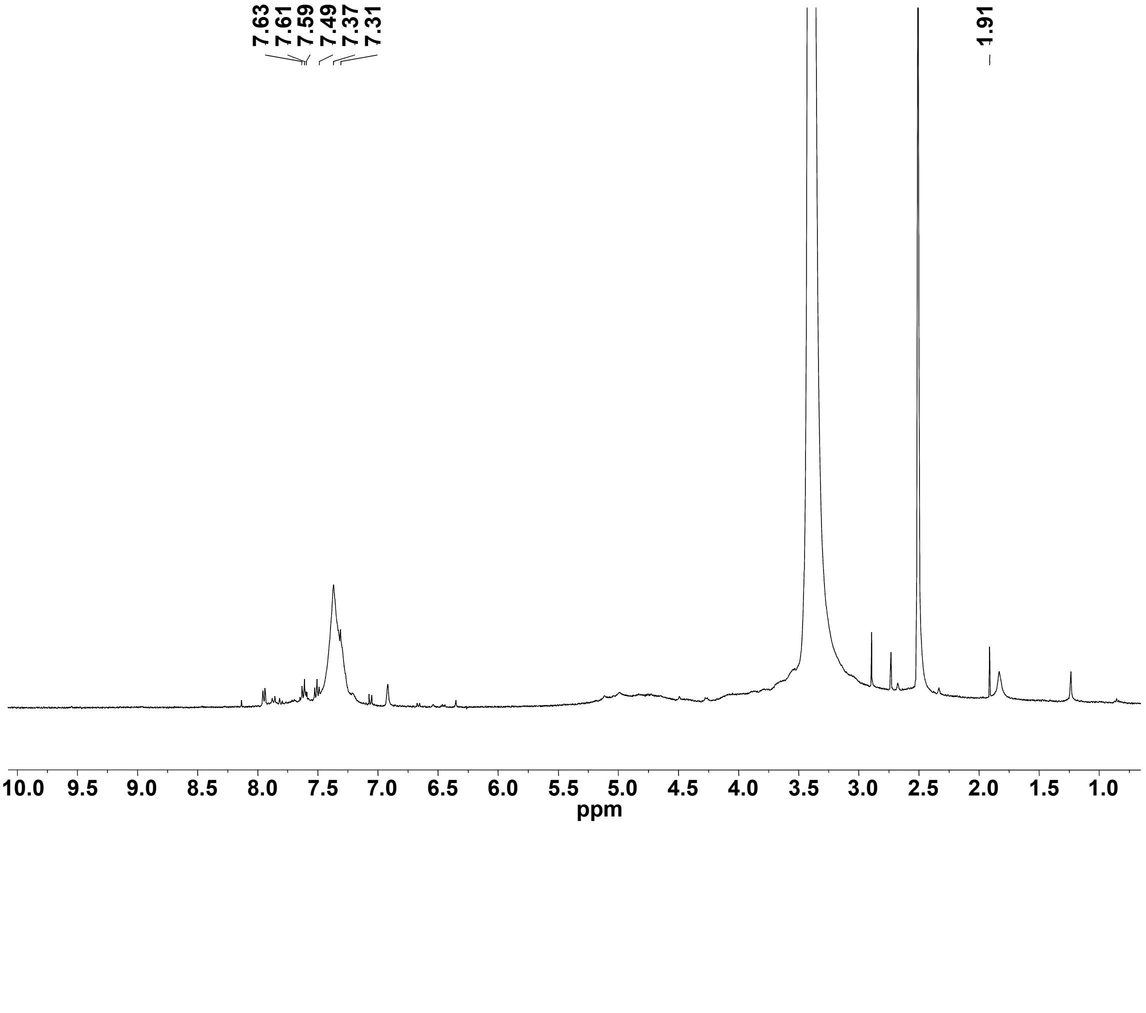

Figure S13. ^{1}H-NMR spectrum of compound **5**.

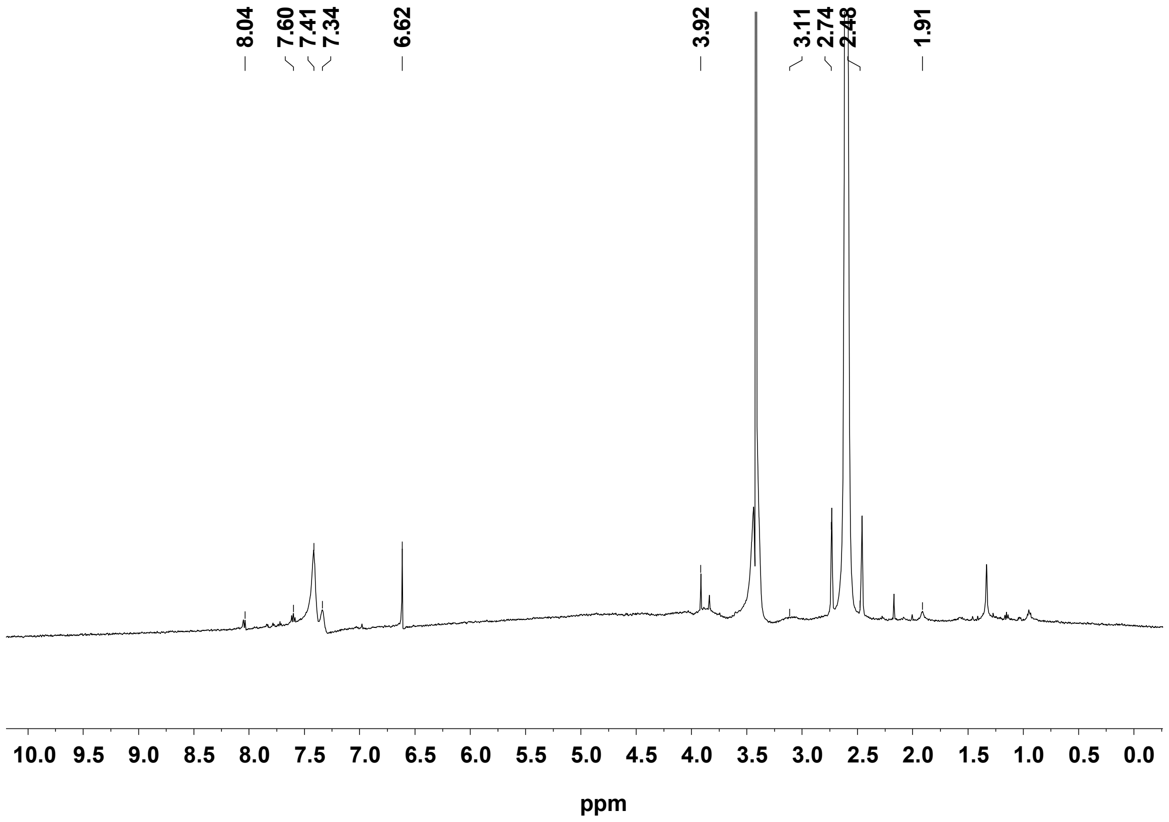

Figure S14. ^{15}N-NMR spectrum of chitosan.

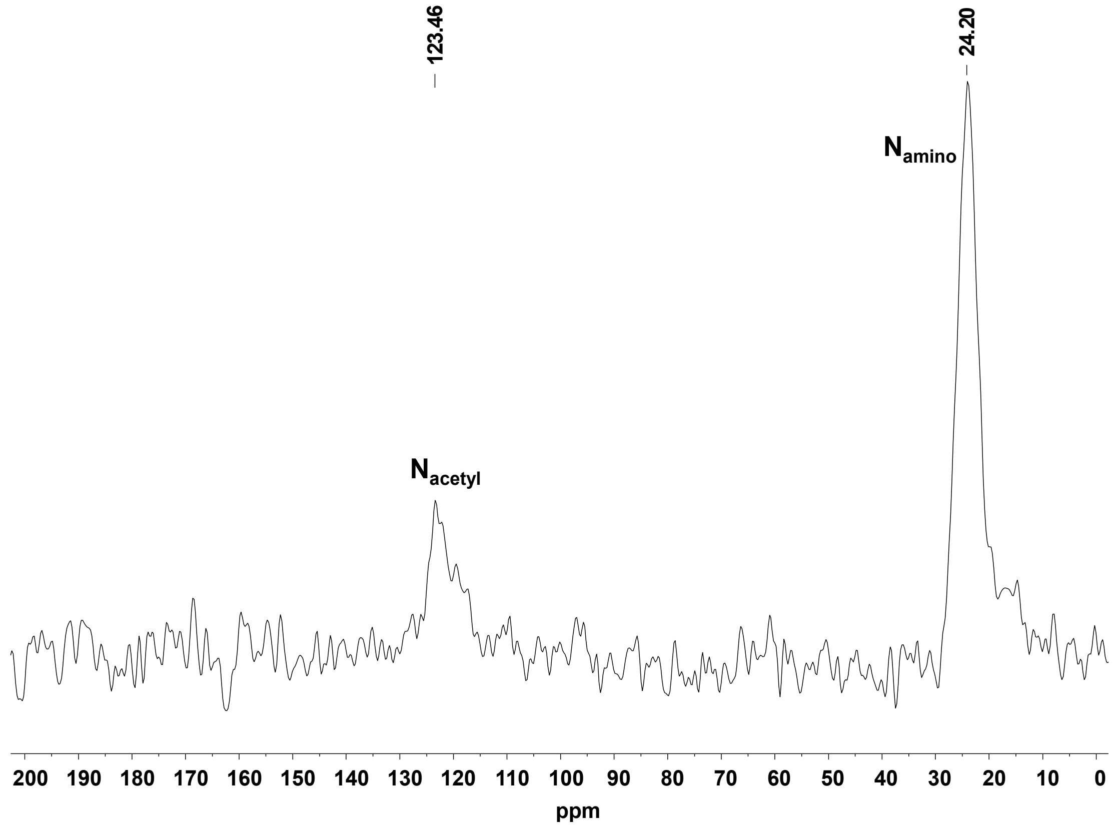

Figure S15. ^{15}N-NMR spectrum of *N*-benzyl chitosan.

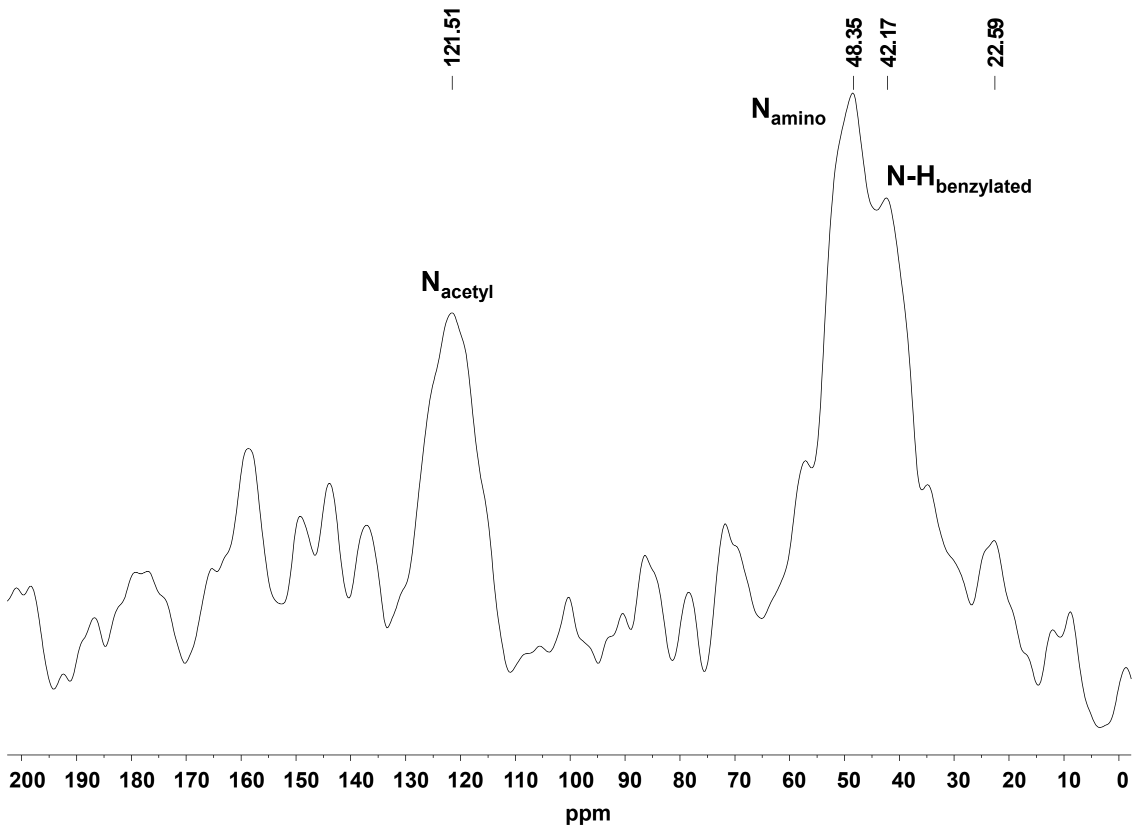

Figure S16. ^{15}N-NMR spectrum of compound **5**.

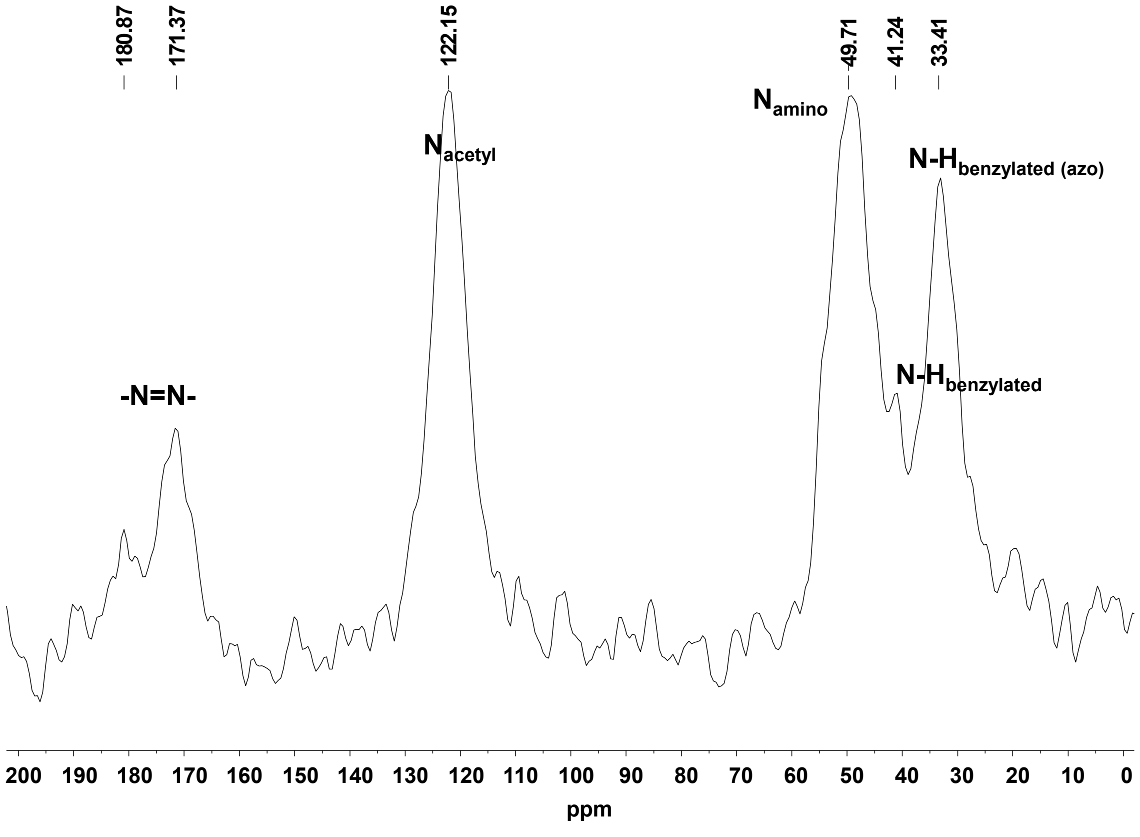

Table S1. Calculated areas for determination of the degree of deacetylation (DD) of chitosan. The ^{15}N-NMR measurements were performed in triplicate.

Signal (ppm)	Chitosan			Average	Error
24.20	2,644,436	2,644,446	2,644,456	2,644,446	10
123.46	707,978	707,988	707,998	707,988	10

Table S2. Calculated areas for determination of the degree of substitution (DS) of *N*-benzyl chitosan. The ^{15}N-NMR measurements were performed in triplicate.

Signal (ppm)	*N*-benzyl Chitosan			Average	Error
42.17	854,556	854,666	854,676	854,666	66
48.35	1,512,335	1,512,325	1,512,345	1,512,325	10
123.46	1,158,029	1,158,039	1,158,049	1,158,039	10

Table S3. Calculated areas for determination of the degree of substitution (DS) of compound **5**. The ^{15}N-NMR measurements were performed in triplicate.

Signal (ppm)	Compound 5			Average	Error
33.41	3,308,711	3,308,701	3,308,722	3,308,701	10
42.17	1,045,387	1,045,397	1,045,498	1,045,397	61
48.35	6,553,375	6,553,475	6,553,585	6,553,485	105
123.46	4,430,778	4,430,788	4,430,798	4,430,788	10
171.37	1,550,330	1,550,340	1,550,350	1,550,340	10
180.87	277,482	277,502	277,522	277,502	20

The degree of deacetylation (DD) and substitution (DS) can be calculated by ^{15}N-NMR as follows:

$$DD = A_{NH2}/(A_{NH2} + A_{N\text{-}acteyl}) \times 100\% \quad (1)$$

A_{NH2} and $A_{N\text{-}acteyl}$ correspond to the integral areas of amino and acetyl groups, respectively.

The DD and DS were calculated using the average value of the areas of each ^{15}N-NMR signal.

Using Equation (1), the DD of chitosan was 78%. The DS of *N*-benzyl chitosan was 56% and compound **5** was 42%.

The degree of deacetylation for chitosan by ^{1}H-NMR technique was determined using the equation:

$$DD\ (\%) = 1 - (1/3\ I_{CH3}/I_{(H2\text{-}GlyN)}) \times 100$$

where I_{CH3} is integral of -CH_3 signal and $I_{(H2\text{-}GlyN)}$ is the integral of the proton of C-2 carbon of GlyN.

The DD value obtained was 78%, which is in agreement with the DD value obtained by ^{15}N-NMR technique.

The degree of substitution of the derivatives was also calculated by ^{1}H-NMR technique using the equation:

$$DS\ (\%) = (Ar/n/I_{H2+H2'} + 1/3\ I_{CH3}) \times 100$$

where Ar is the integral of aromatic protons, n is number of hydrogen atom per substituent, $I_{H2+H2'}$ are integrals of the proton of C-2 carbon of GlyN, and I_{CH3} is the integral of GlyNAc proton.

The obtained DS values are listed in the table below.

Table S4. Degree of substitution (DS) of *N*-benzyl chitosan and compounds **1**–**5**.

Compounds	DS (%)
N-benzyl chitosan	50
1	66
2	31
3	51
4	52
5	46

The integral areas of the proton signals are given in the Tables below.

Table S5. Calculated integrals average for determination of the degree of substitution (DS) of chitosan. The ^{1}H-NMR measurements were performed in triplicate.

Signal (ppm)	Chitosan Integrals Average	Error
1.93	2.43	10
3.06	3.89	10

Table S6. Calculated integrals average for determination of the degree of substitution (DS) of *N*-benzyl chitosan. The ^{1}H-NMR measurements were performed in triplicate.

Signal (ppm)	*N*-benzyl Chitosan Integrals Average	Error
2.00	1.62	50
3.13–3.19	2.97	22
7.36–7.89	6.18	60

Table S7. Calculated integrals average for determination of the degree of substitution (DS) of compound **1**. The ^{1}H-NMR measurements were performed in triplicate.

Signal (ppm)	Compound 1 Integrals Average	Error
1.84	3.10	60
3.54–3.68	13.88	10
7.36–7.89	21.53	10

Table S8. Calculated integrals average for determination of the degree of substitution (DS) of compound **2**. The ^{1}H-NMR measurements were performed in triplicate.

Signal (ppm)	Compound 2 Integrals Average	Error
1.85	1.16	50
3.71–3.76	3.68	22
7.12–8.24	21.53	60

Table S9. Calculated integrals average for determination of the degree of substitution (DS) of compound **3**. The ^{1}H-NMR measurements were performed in triplicate.

Signal (ppm)	Compound 3 Integrals Average	Error
1.87	1.03	38
3.61–3.67	4.11	42
7.32–8.38	18.40	10

Table S10. Calculated integrals average for determination of the degree of substitution (DS) of compound **4**. The ^{1}H-NMR measurements were performed in triplicate.

Signal (ppm)	Compound 4 Integrals Average	Error
1.83	5.35	10
3.053–3.16	10.41	10
7.25–8.24	54.50	10

Table S11. Calculated integrals average for determination of the degree of substitution (DS) of compound **5**. The ^{1}H-NMR measurements were performed in triplicate.

Signal (ppm)	Compound 5 Integrals Average	Error
1.92	1.30	40
3.83–3.92	4.64	34
7.33–7.60	15.64	67

Figure S17. UV-Vis spectra of compounds **1**–**5**.

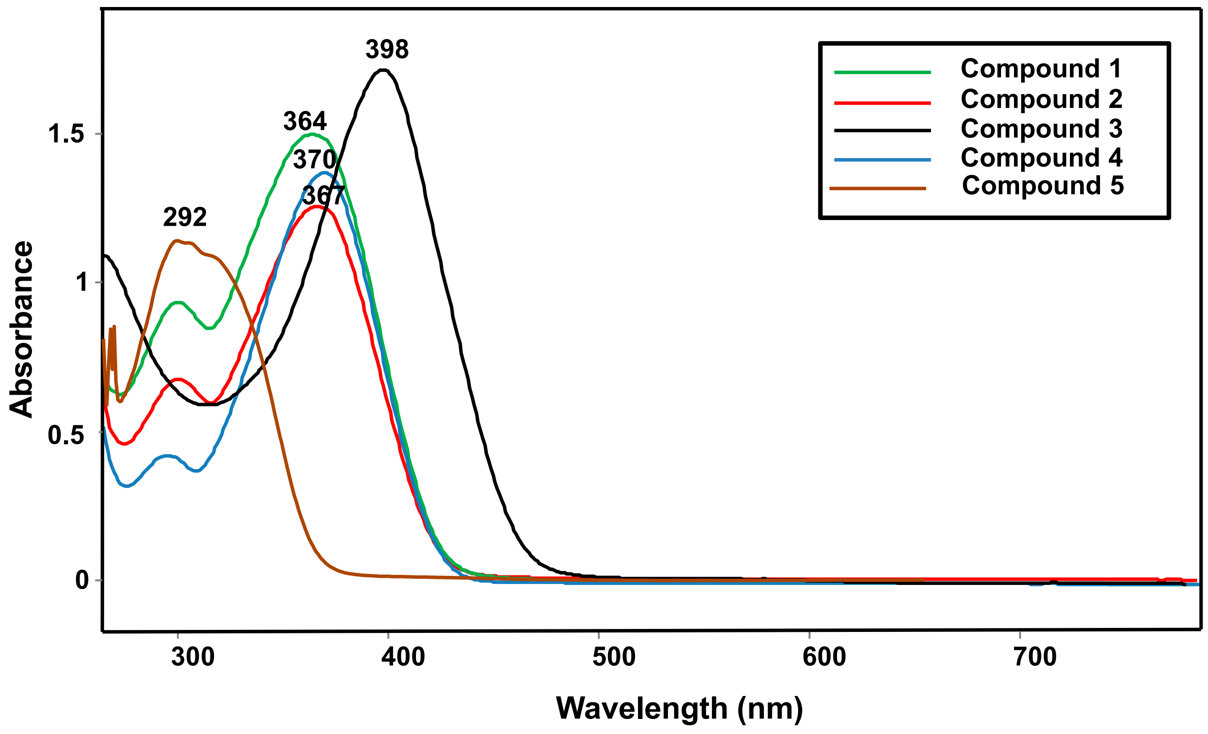

Figure S18. ESI(+)MS spectrum of chitosan hydrolysates.

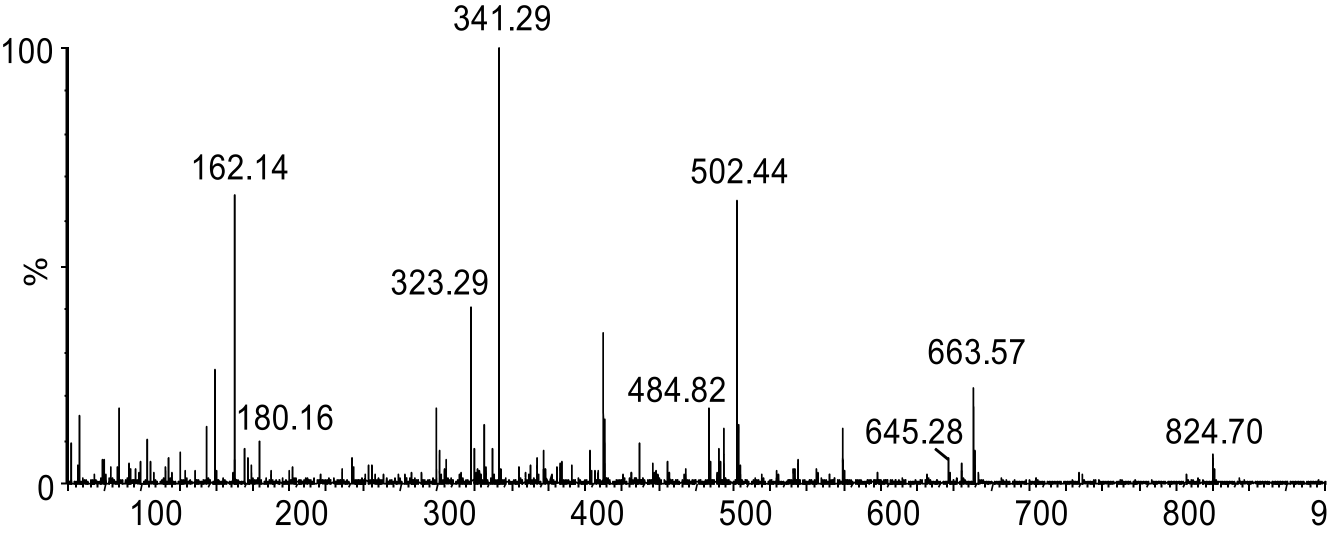

Figure S19. ESI(+)MS spectrum from the hydrolysis reaction mixture of compound **4** (10 M HCl).

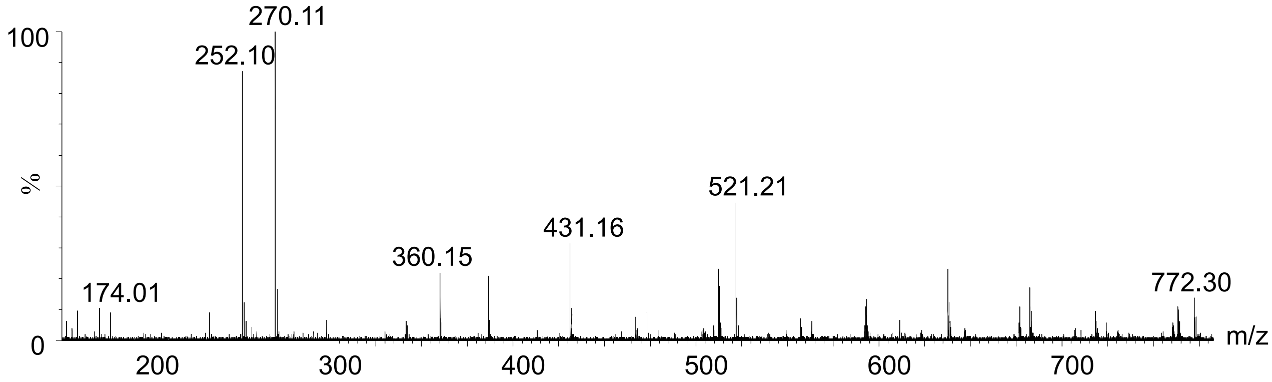

Figure S20. ESI(+)MS spectrum from the hydrolysis reaction mixture of compound **5** (10 M HCl).

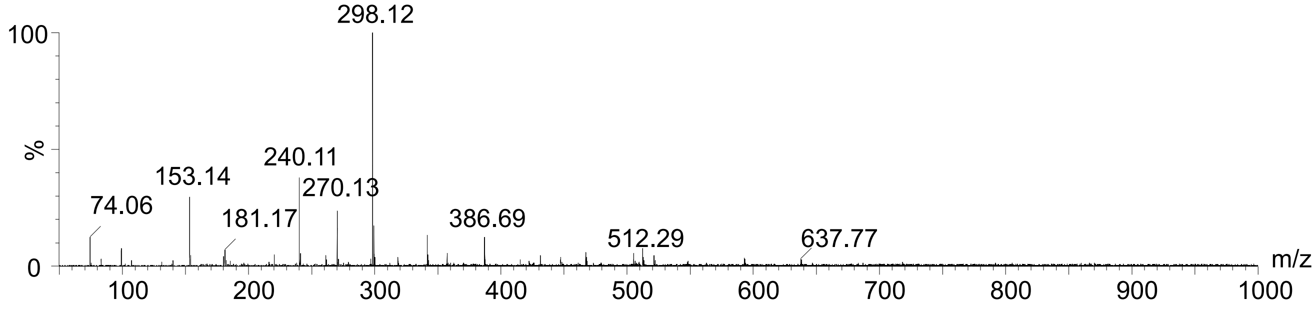

Figure S21. GC-MS spectrum from the hydrolysis reaction mixture of *p*-bromoaniline (10 M HCl).

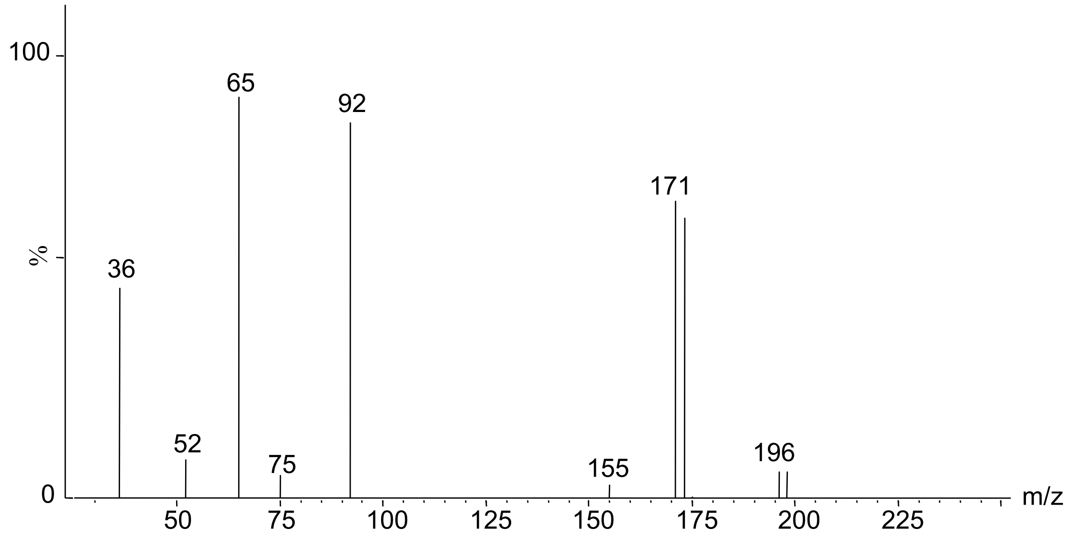

Figure S22. GC-MS spectrum from the hydrolysis reaction mixture of *p*-bromobenzenediazonium tetrafluorborate (10 M HCl).

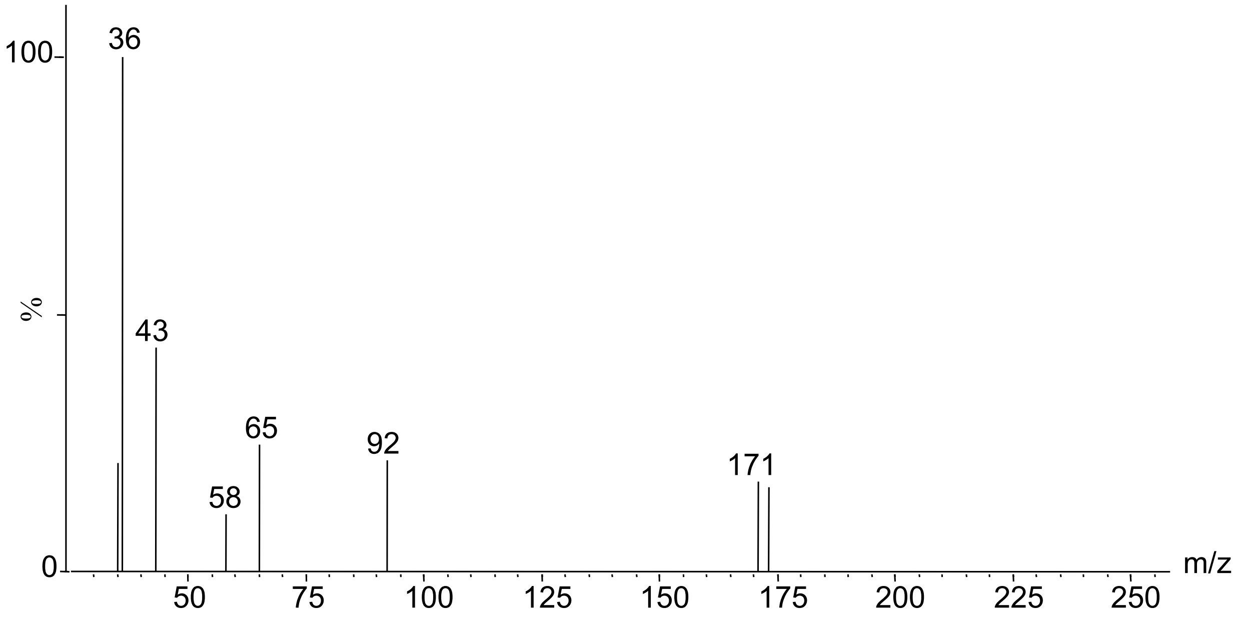

Supplement: Supplementary File 1 [file molecules-19-17604-s001.pdf]
